# Psychosis in Parkinson’s Disease: A Lesson from Genetics

**DOI:** 10.3390/genes13061099

**Published:** 2022-06-20

**Authors:** Efthalia Angelopoulou, Anastasia Bougea, Sokratis G. Papageorgiou, Chiara Villa

**Affiliations:** 1 Department of Neurology, Eginition University Hospital, National and Kapodistrian University of Athens, 11528 Athens, Greece; angelthal@med.uoa.gr (E.A.); abougea@med.uoa.gr (A.B.); sokpapa@med.uoa.gr (S.G.P.); 2 School of Medicine and Surgery, University of Milano-Bicocca, 20900 Monza, Italy

**Keywords:** psychosis, Parkinson’s disease, genetics

## Abstract

Psychosis in Parkinson’s disease (PDP) represents a common and debilitating condition that complicates Parkinson’s disease (PD), mainly in the later stages. The spectrum of psychotic symptoms are heterogeneous, ranging from minor phenomena of mild illusions, passage hallucinations and sense of presence to severe psychosis consisting of visual hallucinations (and rarely, auditory and tactile or gustatory) and paranoid delusions. PDP is associated with increased caregiver stress, poorer quality of life for patients and carers, reduced survival and risk of institutionalization with a significant burden on the healthcare system. Although several risk factors for PDP development have been identified, such as aging, sleep disturbances, long history of PD, cognitive impairment, depression and visual disorders, the pathophysiology of psychosis in PD is complex and still insufficiently clarified. Additionally, several drugs used to treat PD can aggravate or even precipitate PDP. Herein, we reviewed and critically analyzed recent studies exploring the genetic architecture of psychosis in PD in order to further understand the pathophysiology of PDP, the risk factors as well as the most suitable therapeutic strategies.

## 1. Introduction

Parkinson’s disease (PD) is a chronic and progressive neurodegenerative movement disorder associated with progressive disability and characterized by both motor and non-motor symptoms [1]. PD represents the second most common age-associated neurodegenerative disorder after Alzheimer’s disease (AD) [2]. Patients experience motor features, including resting tremor, bradykinesia and muscular rigidity with postural instability, often appearing as the disease progresses [3]. Pathologically, these symptoms are mostly attributed to the extensive degeneration of striatal dopaminergic neurons in the *substantia nigra pars compacta* (SNpc) projecting to the dorsal striatum [4], resulting in a loss of dopamine transmission throughout the brain. At the histological level, the progressive SNpc degeneration correlates with the accumulation of large intra-cytoplasmic inclusions, namely Lewy bodies (LBs) containing misfolded α-synuclein (α-syn), neurofilaments and ubiquitin [5], although α-syn deposition occurs years before motor presentations begin. PD patients also suffer from non-motor symptoms, such as autonomic dysfunction, pain, olfactory deficits, sleep disorders, cognitive impairment and psychiatric disturbances [6]. The underlying mechanisms of PD-related non-motor manifestations are far less clear than motor features and still very difficult to treat.

Among the different non-motor symptoms of PD, psychosis in PD (PDP) is one of the most common, complex and disabling non-motor features, with an estimated prevalence of 43–63% in later stages of the disorder [7,8,9]. PDP prevalence increases with disease progression and it is associated with poorer quality of life, disability and caregiver stress, as well as accelerated cognitive decline, hospitalization or institutionalization, morbidity and mortality [10]. Importantly, the clinical features observed in PDP have a different pattern as compared to other psychotic diseases such as schizophrenia or mood disorders associated with psychotic phenomena, so the current diagnostic criteria applied to other psychiatric illnesses may be unsatisfactory when describing the diversity of PDP [11]. The spectrum of psychotic symptoms experienced by PD patients consist of hallucinations (mainly visual, but also auditory, tactile or gustatory) and delusions, which simplistically define psychosis. Additionally, there are also minor psychotic phenomena, which include passage hallucinations, sense of presence and illusions [12]. Once psychotic features develop, they tend to become progressive and persistent. Although PDP has some common mechanisms to other psychotic disorders, the neurobiology is different, complex and still insufficiently known [11]. Neuroimaging and neuropathological studies have implicated executive function and visual processing deficits in neocortex and limbic structures, with an imbalance between dopamine, acetylcholine and serotonin neurotransmission [13]. Moreover, PDP therapeutic strategies still continue to be a challenge as dopaminergic treatment for PD motor symptoms, such as levodopa or dopaminergic agonists, exacerbates the condition [8] and the administration of antipsychotic drugs in vivo have revealed a high rate of mortality and morbidity [14]. Apart from exogenous factors, including dopaminergic treatment, several intrinsic factors have been associated with PDP development, including ageing, a more advanced stage of the disease, depression, cognitive impairment, female sex and REM sleep behavior disorder and daytime sleepiness [13,15]. However, not all PD patients develop psychosis, and dopaminergic drugs only partially contribute to the PDP risk. PDP has also been observed in drug naïve PD patients [16]. Although an increasing number of studies has investigated the relationship between several genetic factors and psychotic symptoms in PD, their role in PDP is still unclear.

Since PD is a clinically and molecularly heterogeneous disorder, the elucidation of clinical-genetic associations may help us to clarify its pathogenesis and allow for more personalized preventive, prognostic and treatment plans. There is a need for biomarkers that could characterize PD phenotypes. Although the genetic factors associated with psychotic symptoms in PD have been already discussed elsewhere [17], there is no comprehensive review incorporating and critically analyzing recent evidence during the last five years. Given the significant impact of psychosis on PD, this review aims to discuss the genetic architecture of PDP in order to further understand the pathophysiology of psychotic symptoms, especially in idiopathic PD, the risk factors and the most suitable therapeutic strategies.

## 2. Methods

We searched “PubMed” and “Scopus” databases for research and review articles investigating the role of genetic factors in PD-related psychosis. Although our aim was not to conduct a systematic review, we used the following keywords: “genes”, “genetic”, “SNPs”, “gene polymorphisms”, “Parkinson’s disease psychosis”, “PD”, “psychotic”, “psychosis”, “hallucinations” and “delusions” in different combinations, in order to ensure that the majority of relevant articles have been identified. Articles written in the English language were selected because of their relevant titles and abstracts. We also screened the bibliography of each relevant article for other possible relevant studies. No specific inclusion or exclusion criteria were set. We summarized and synthesized the findings from the relevant articles in two tables, extracting and documenting their key findings.

## 3. The Genetic Landscape of Parkinson’s Disease: A Brief Overview

The vast majority of PD cases are idiopathic (also defined as sporadic or sometimes “non-genetic”) with a multifactorial etiology, whereas only approximately 5–10% are the so-called monogenic forms (sometimes called Mendelian, familial or genetic), caused by pathogenic variants in single genes inherited with Mendelian transmission pattern [18,19] (summarized in Table 1).

### 3.1. Monogenic Forms of PD

In the late 1990s, the analysis of a large multigenerational Italian kindred and three unrelated Greek families led to the identification of the first PD-related missense mutation in the *SNCA* gene (*PARK1*/*PARK4*), encoding for α-syn [20]. In the subsequent twenty years, many additional point mutations and whole gene multiplications of *SNCA* have been described, further supporting a key role of α-syn misfolding and aggregation in the disease pathogenesis [21]. Although missense mutations in *SNCA* gene are quite rare, duplications or even triplications of normal *SNCA* have been largely detected in several PD cases [22]. Moreover, the number of *SNCA* copies has been found to correlate with a more severe clinical phenotype and rapid progression, suggesting a dosage effect [22,23]. PD displays a great genetic heterogeneity, with many different rare mutations often found only in small populations or in a single family [20].

Besides *SNCA*, additional well-established genes are linked with monogenic forms of the disease: autosomal dominant PD is caused by mutations in leucine-rich repeat kinase 2 (*LRRK2*/*PARK8*) and vacuolar sorting protein 35 (*VPS35*/*PARK17*) genes, whereas mutations in *PRKN* (*Parkin*/*PARK2*), PTEN-induced putative kinase 1 (*PINK1*/*PARK6*) and protein deglycase (*DJ-1*/*PARK7*) genes are associated with autosomal recessive PD [18,24]. In contrast to *SNCA* mutations that are rare, pathogenic variants detected in *LRKK2* gene represent the most commonly frequent causes of PD, with an estimated frequency of 5% and 1% in familial and sporadic cases, respectively [25,26]. *LRRK2* encodes for a large and multifunctional protein characterized by dual kinase and GTPase activity, involved in neurite outgrowth, neuronal vesicular trafficking and autophagic protein degradation [27,28]. Therefore, mutations in this gene may exert their pathogenic effect via a gain-of-function mechanism, probably by increasing the kinase activity [29]. Another PD-related gene inherited with an autosomal dominant pattern is *VPS35*, encoding a component of the retromer complex which is responsible for the recycling of membrane proteins from endosomes to the trans-Golgi network, as well as for the degradation of mitochondrial proteins through the formation of mitochondria-derived vesicles [30]. *VPS35* mutations are supposed to alter the stability and intracellular localization of these organelles [31].

Established genes associated with recessive forms of PD are *PRKN*, *PINK1* and *DJ-1*, which share the same cellular pathway, playing a key role in mitochondrial homeostasis and selective clearance of dysfunctional mitochondria via the autophagy/lysosome pathway [32]. Among these genes, *PRKN* encodes Parkin, a component of the E3 ubiquitin ligase complex involved in the proteasome-mediated degradation of damaged proteins and mitophagy [33]. Mutations in this gene represent the most common cause of autosomal recessive early-onset PD and are highly variable, including structural variants (multiplications and deletions in exons or gene promoter), missense, nonsense, splice-site and frameshift mutations, detected in homozygous or compound-heterozygous states [34]. In all cases, these pathogenic variants result in loss of Parkin function through different mechanisms [35,36]. The second common gene responsible for autosomal recessive forms of PD is *PINK1*, which encodes a mitochondrial serine–threonine kinase able to trigger the phosphorylation-dependent activation of Parkin, maintaining its mitochondrial stabilization and translocation to damaged mitochondria [37,38]. The majority of mutations in *PINK1* induce conformational changes in its catalytic domains leading to loss of kinase activity and the subsequent block of Parkin translocation for mitochondrial quality check [39]. After the *PRKN*- and *PINK1*-related cases, biallelic variants in *DJ-1* have been linked to a rare form of autosomal recessive PD with early onset. DJ-1 was shown to be involved in many cellular processes, including autophagy, apoptosis regulation, protection against oxidative stress by scavenging reactive oxygen species and acting as molecular chaperones to promote correct protein folding [40]. The PD-related loss of DJ-1 function is associated with increased sensitivity to oxidative stress-induced cell death, reduced basal autophagy and accumulation of damaged mitochondria [41].

### 3.2. Sporadic Forms of PD

Whereas the monogenic forms of PD have been originally identified through linkage analysis in families with several affected members, genetic risk factors associated with sporadic PD have been detected by candidate gene analyses and genome-wide association studies (GWAS). Risk variants underlying susceptibility to sporadic PD are often heterogeneous and vary in frequency according to the genetic background of each ethnic group, further supporting the complexity of PD genetics [42]. GWAS studies identified several PD-related loci, but the most robust and consistently replicated risk factors have been detected in *SNCA*, *LRRK2*, *MAPT* and *GBA* [43,44,45]. Interestingly, variants in some disease-causing genes have been also found to increase the risk of developing sporadic PD, suggesting a common neurodegenerative process between familial and sporadic PD [42,46]. This is the case for *SNCA* and *LRRK2*, in which rare coding mutations (with a high effect size) in these genes cause monogenic PD forms whereas more common variants (with a smaller effect size and often in non-coding regions) can also confer the risk for developing the disorder [32,46].

With regards to *SNCA*, it has also been recently reported that single nucleotide polymorphisms (SNPs) located in non-coding regions may play a role in gene expression regulation by post-transcriptional modifications interfering with alternative splicing mechanisms or interacting with microRNAs [46]. Additionally, functional studies revealed that the allele-length variability in the dinucleotide repeat sequence (REP1) of the *SNCA* gene promoter increases sporadic PD susceptibility [47,48]. Mimicking *SNCA* locus multiplication, the 261 bp-long risk allele was associated with α-syn upregulation, whereas the 259 bp-long protective allele decreased gene expression [47]. However, *SNCA* represents a low-risk locus for idiopathic PD with odds ratios (ORs) ranging from 1.2 to 1.4, as demonstrated by meta-analyses [49].

Variants in the *LRRK2* gene have been frequently associated with increased risk for developing sporadic PD. As stated above, besides the prevalence of p.Gly2019Ser in rare monogenic PD, this mutation can also increase the risk for the idiopathic forms depending on age and ethnic background [26,50]. The p.Gly2019Ser is very rare in Asians whereas the p.Gly2385Arg and p.Arg1628Pro variants are more frequent in Asian populations than in Caucasians with an estimated OR of 2.2 and 1.84, respectively [51,52].

Despite the fact that *MAPT* has been frequently associated with tauopathies such as AD and frontotemporal dementia [53,54], SNPs in this gene represent risk factors for α-synucleinopathy as well, suggesting possible shared pathogenic mechanisms among different neurodegenerative disorders. *MAPT* encodes the microtubule-associated protein tau which is essential for microtubule assembly and stabilization, normal axonal transport and neurite outgrowth [55]. There are two common haplotypes spanning the entire *MAPT* coding region ensued from an ancestral inversion of 900 kb on chromosome 17q21: the directly oriented H1 and the inverted H2 [56]. Whereas the H1 haplotype is common in all populations, the H2 haplotype is thought to be exclusively present in populations of Caucasian origin, rare in Africans and nearly absent in Americans and East Asians [57]. Data from GWAS studies and meta-analyses supported the common *MAPT* H1 haplotype as a susceptibility factor for sporadic PD with an OR of approximately 1.5 [49,58,59,60].

Biallelic (homozygous or compound heterozygous) mutations in *GBA* gene, encoding the enzyme glucocerebrosidase (GCase), have been detected in patients affected by Gaucher disease (GD), a rare lysosomal storage disorder inherited with an autosomal recessive pattern [61]. Clinical evaluation of GD phenotypic heterogeneity has revealed the occurrence of parkinsonian features in patients with GD and among their relatives [62,63]. Variants harboring *GBA* gene have been found in approximately 8.5% of sporadic PD cases [64], representing the principal genetic risk for this disease until now [65]. However, the frequency of *GBA* mutations in PD varies significantly across different ethnicities, being more common in the Ashkenazi Jewish population, with about a five-fold increased risk to develop PD [65,66]. Additionally, carriers of *GBA* variants also display an earlier disease onset (<50 years), a three-fold increased risk to develop dementia and a more rapid disease course compared to non-carriers [67,68]. Despite extensive research, the molecular and cellular mechanisms underlying the high PD risk observed in carriers of *GBA* mutations remain to be elucidated [69].

### 3.3. X-Linked PD

Pathogenic variants in the gene encoding the small GTPase protein, Ras Analogue in Brain 39b (RAB39B), were initially identified in a few families affected by non-progressive intellectual disability in comorbidity with macrocephaly, autism spectrum disorders and epilepsy [70,71]. More recently, additional evidence reported that loss-of-function mutations in *RAB39B*, including whole gene deletion, missense, splicing and frameshift variants, also led to the development of an X-linked form of rare early-onset PD associated with intellectual disability [72,73]. RAB39B is a neuronal protein which is localized in the Golgi compartment and acts as an essential regulator of vesicular trafficking, possibly affecting α-syn homeostasis [74]. This protein is also believed to play a role in the modulation of growth cone function, neurite morphology, synaptic plasticity and synapse formation [74,75]. However, studies in large cohorts of PD patients failed to detect pathogenic variants in *RAB39B*, suggesting that mutations in this gene represent a very rare cause of PD [76,77].

### 3.4. Other Disease-Causing Genes

In addition to the well-established genes associated with Mendelian forms of PD, a number of other candidates have been later suggested as putative disease-causing, albeit limited, evidence for their pathogenicity [78]. Among them, biallelic rare mutations in *DNAJC6* and *SYNJ1*, encoding interacting proteins involved in synaptic vesicle endocytosis and recycling, have been found in patients affected by early-onset PD with autosomal recessive inheritance. In addition, DNAJC13 and TMEM230 are implicated in synaptic vesicle processing, but mutations in their encoding genes have been identified in the same Canadian family with PD, suggesting their questionable pathogenicity [79]. Subsequent studies failed to replicate the association of *TMEM230* with PD, thus casting doubt upon its involvement in disease pathogenesis [18].

A GWAS study performed in a large Italian pedigree with members affected by autosomal dominant PD and dementia with Lewy bodies (DLB) led to the identification of heterozygous mutations in the gene encoding low-density lipoprotein receptor-related protein 10 (*LRP10*) [80]. However, replication studies in different ethnicities failed to confirm this association [18].

Whole-exome sequencing performed in the Han Chinese population identified *NUS1* as possible PD-causing gene [81]. The same authors validated the presence of rare damaging nonsynonymous variants in two large independent case-control cohorts. Functional studies performed in *Drosophila* further revealed that *NUS1* loss could decrease dopamine levels, climbing ability and the number of dopaminergic neurons as well as activate apoptosis in fly brain [81], thus strengthening a possible involvement of *NUS1* in PD pathogenesis. This gene encodes the Nogo-B receptor (NgBR), a type I single transmembrane domain receptor, which is a subunit of cis-prenyltransferase essential for the biosynthesis of dolichol and protein glycosylation [82]. It is also involved in the trafficking of low-density lipoprotein-derived cholesterol through the interaction and stabilization of the Niemann-Pick type C2 protein (NPC2) [83]. A subsequent case-control study in Europeans failed to replicate previous data, suggesting that the observed association between *NUS1* variants and PD may be related to hugely rare population-specific variants [84].

In a family with a history of PD, some authors recently discovered two compound heterozygous missense mutations in the arylsulfatase A (*ARSA*) gene, encoding a lysosomal hydrolase, further supporting the role of lysosomal system in the disease pathogenesis [85]. Later, both in vitro and in vivo studies revealed that ARSA acts as a cytosolic molecular chaperone interacting with α-syn, regulating its secretion, accumulation and cell-to-cell propagation [86]. However, large international consortiums did not confirm the link between *ARSA* and PD [87].

## 4. Genetic Architecture of Psychosis in Parkinson’s Disease

### 4.1. Potential Association between APOE Genotype and PDP

Apolipoprotein E (APOE) is a lipoprotein abundantly found in the brain, being implicated in several cellular processes including the metabolism and clearance of β-amyloid [88]. *APOE* gene has three alleles, named ε2, ε3 and ε4. *APOE* ε3 is the most frequent allele, whereas the ε4 allele is the most common genetic risk factor for AD, leading to an earlier onset of the disease as well [88]. Furthermore, *APOE* ε4 has been linked to hallucinations and delusions in AD patients [89], and may be also an age-related risk factor for the worsening of delusions and hallucinations in patients with schizophrenia [90]. In PD, the *APOE* ε4 allele has been also associated with earlier disease onset [91,92] and a greater risk of dementia [93], although there is also evidence that does not confirm these relationships [92,94]. Furthermore, *APOE* ε4 has been associated with increased severity of Lewy body pathology independent of AD pathology in another study, suggesting that *APOE* ε4 may promote the spreading of LBD [95]. Moreover, hallucinations have been shown to be more common in patients with dementia with LBs carrying *APOE* ε4 [96]. Given the well-established association between psychosis and dementia in PD [97], as well as the relationship between advanced disease and the manifestation of psychotic symptoms, it has been suggested that *APOE* ε4 may increase the risk of PDP.

In this context, the presence of the *APOE* ε4 allele has been associated with higher risk of visual hallucinations in PD patients without dementia treated with levodopa [98]. On the contrary, another four studies did not confirm this relationship [16,99,100,101], one of which used autopsy-confirmed PD cases [100]. However, since PDP risk increases as the disease progresses, the role of the *APOE* ε4 allele in modifying the age at onset of psychotic symptoms in PD would be of particular interest. Indeed, another study demonstrated that carrying the *APOE* ε4 allele was correlated with earlier development of psychosis in PD patients, independently of cognitive status or age [102]. In accordance, it has been demonstrated that the development of hallucinations during the first five years of the disease was significantly more common in PD patients carrying the *APOE* ε4 allele, compared to those not carrying this allele [103]. On the other hand, *APOE* ε4 was not associated with the occurrence of hallucinations five years after PD diagnosis in this study [103]. Collectively, it seems that *APOE* ε4 may be associated with an earlier onset of hallucinations in PD, and maybe during the early stages of the disease especially. However, the replication of this evidence in longitudinal studies with larger sample sizes is required.

### 4.2. Dopamine Transporter (DAT) Gene Polymorphisms and PDP Development

Dopamine transporter (DAT) modulates the reuptake of dopamine in the presynaptic dopaminergic neurons, being highly involved in the temporal and spatial regulation of dopamine recycling [104]. VNTR is the most commonly studied polymorphism in the *DAT* gene, located in the non-coding 3′ untranslated region (3′ UTR), consisting of 40 base pairs (bp), which are repeated from three to eleven times. The most common alleles are those with nine and ten repeats, whereas the number of repeats might regulate gene expression and the subsequent retrieval of dopamine in the synapsis [17]. *DAT* polymorphisms have been linked to neuropsychiatric diseases, including bipolar disorder and attention deficit hyperactivity disorder [104]. In addition, the A9 allele of the *DAT* gene increases the risk of visual hallucinations in alcohol-dependent women during alcohol withdrawal [105]. The 40-bp VNTR of the *DAT* gene has been associated with increased susceptibility to PD [106,107,108].

*DAT* gene polymorphisms have been associated with a greater frequency of PDP in some but not all studies. More specifically, the nine copy allele of the 40-bp VNTR of the *DAT* gene was more commonly identified in levodopa-treated PD patients of Caucasian descent with psychosis compared to those without psychosis [109]. Preclinical evidence has demonstrated that compared to the ten-copy allele of the *DAT* gene, the nine-copy allele could promote the transcription of the gene, even though it is located in a non-coding region [110]. Therefore, it has been hypothesized that the nine-copy allele-mediated enhanced *DAT* gene expression and subsequent higher DAT levels might increase dopamine reuptake and lead to cellular damage [109]. On the other hand, another recent study in Serbia did not find significant associations between the 40-bp VNTR of the *DAT* gene and PDP [111]. This study included PD patients not only with visual hallucinations but also other psychotic symptoms, such as delusions. In accordance, another study also failed to reveal any significant associations between the VNTR of *DAT* and PDP [112]. No associations between visual hallucinations in levodopa-treated PD patients and the 40-bp VNTR of the *DAT* gene have been demonstrated in another study in China [113], highlighting the crucial factor of ethnicity in determining the influence of DAT polymorphisms on PDP. Indeed, Caucasian and Chinese populations display variations in the 40-bp VNTR of the *DAT* gene [113], highlighting the possible effects of ethnic backgrounds on PD. In addition, methodological differences, mainly in regard to the definition of PDP (the inclusion of patients with visual hallucinations only or/and delusions) may have contributed to these observed conflicting results.

Further, the −839 C > T allele of the 5’ UTR of the *DAT* gene has been associated with visual hallucinations in levodopa-treated PD patients [112]. It has been speculated that this polymorphism may affect *DAT* gene expression, although its exact functional consequences are unclear [112]. Concerning *DAT1* rs28363170, a recent study in Brazilian PD patients showed that carrying the 10/11, 10/8 and 10/9 genotypes was more frequently associated with visual hallucinations, but no association was found for *DAT1* rs28363170 [114]. In addition, a recent study in Slovenia demonstrated that the haplotype of the *SLC6A3* gene was associated with the development of levodopa-induced visual hallucinations in PD patients [115]. Taken together, the role of *DAT* genes in PDP still remains obscure.

### 4.3. Dopamine Receptor (DRD) Gene Polymorphisms and the Risk for PDP

Dopaminergic therapy is one of the most significant risk factors for PDP; hence, dopaminergic receptor-related pathways might be an appropriate target for exploring PDP pathophysiology. Dopaminergic agonists act by directly stimulating the postsynaptic dopamine receptors in the striatum, with a relative selection for the dopamine D2-like receptors, including DRD2, DRD3 and DRD4, compared to dopamine D1-like ones, including DRD1 and DRD5 [116]. The use of dopaminergic agonists significantly increases the risk for PDP; in particular, apomorphine, which has a higher affinity for DRD4 compared to DRD2 and DRD3, displays modest hallucinogenic effects, and pergolide, which has greater affinity for DRD2 and DRD3 compared to DRD4, is significantly associated with hallucinations [117]. Therefore, the tendency of dopaminergic agonists to induce psychotic symptoms may be DRD2- and DRD3-mediated. In addition, neuroleptic drugs used for the treatment of schizophrenia mainly target against DRD2. In PD, the neurodegenerative process leads to a denervation-induced hypersensitivity, and DRD2 upregulation has been demonstrated in vivo [116]. DRD2 is found in various brain areas including the striatum and limbic system, whereas DRD3 and DRD4 are mainly located in the limbic system [117]. It has been suggested that hypersensitivity of the dopaminergic mesocorticolimbic system could be implicated in the development of hallucinations in PD, especially those early in the course of the disease not generally related to cognitive decline [117].

*DRD* gene polymorphisms have already been investigated for schizophrenia and AD-related psychosis. In particular, the *DRD3* Ser-9 allele has been associated with higher risk for schizophrenia [118], although there is also evidence not confirming this hypothesis [119]. In AD, psychotic symptoms and aggressive behavior have been associated with homozygosity for *DRD1* B2, whereas *DRD3* 1/1 or 2/2 homozygotes AD patients were more likely to develop psychosis [120]. In addition, the risk for psychotic manifestations was higher in the case of co-presence of *DRD1* and *DRD3* polymorphisms compared to these gene polymorphisms alone in this study, suggesting a possible interaction. There is also evidence that in PD, the *DRD3* gene Ser9Gly polymorphism may be associated with the severity of depressive symptoms in PD patients [121].

Based on this evidence, several studies have explored the role of both classes of DRDs in PDP development. However, relative existing evidence is rather conflicting. *DRD2* (141C/del and TaqIA) and *DRD3* (Ser9Gly) polymorphisms were not associated with dopaminergic therapy-related hallucinations in PD Caucasian patients [117]. Although not statistically corrected for multiple comparisons, further subgroup analysis revealed that the C allele of the TaqIA polymorphism of the *DRD2* gene was more frequently present in PD patients with late-onset hallucinations, defined as those presented five years after the onset of the disease [117]. The authors proposed that the greater extent of denervation hypersensitivity at the later stages of the disease might explain the absence of an association at earlier stages of PD. These findings suggest that this gene polymorphism might be in linkage disequilibrium with a *DRD2* mutation or a mutation in gene close to *DRD2*, which could increase the risk of dopaminergic therapy-related hallucinations in later stages of PD [117]. *DRD2*, *DRD3* and *DRD5* gene polymorphisms have not been associated with visual hallucinations in PD in a study in the Chinese population [113]. Another study also didn’t find any significant associations between *DRD2*, *DRD3* or *DRD4* gene polymorphisms and levodopa-related psychosis in PD Caucasian patients [109]. Furthermore, no association has been demonstrated between *DRD1*, *DRD3* and *DRD4* gene polymorphisms and the presence of hallucinations in PD patients in a center in Chicago, after adjustment for age and dopaminergic medication [99]. However, the frequency of the *DRD3* 2 allele was only marginally increased in PD patients with hallucinations (*p* = 0.05) [99]. Another Brazilian study demonstrated that PD patients with the *DRD3* Ser/Ser and Ser/Gly genotypes of rs6280 displayed higher frequency of visual hallucinations, but no associations were found for *DRD1* rs4532 and *DRD2* rs1800497 [114]. Another study demonstrated that the allele G at *DRD1* A–48G in PD patients was associated with higher risk for visual hallucinations throughout the disease course. In addition, visual hallucinations occurred significantly earlier in PD patients carrying *DRD1* –48 GG and 62TT alleles in this study, whereas *DRD4* CG alleles of 747,302 were associated with later onset of hallucinations, compared to *DRD4* CC alleles [122]. In addition, the TT rs6277 *DRD2* gene variant has been associated with PDP in levodopa-treated PD patients in a study in Serbia [111]. A recent study in Slovenia indicated that PD patients carrying at least one *DRD3* rs6280 C allele and CC homozygotes displayed more frequently levodopa-induced visual hallucinations compared to those not carrying this allele [115].

This partially conflicting evidence could be explained by methodological differences, including the definition of visual hallucinations. Some studies have included patients with “significant hallucinations”, defined as those necessitating medical intervention [122] but, in this case, milder symptoms could have been missed. Ethnic discrepancies may also play a significant role, given the fact that ethnicity has been shown to determine the impact of *DRD* gene polymorphisms in some other conditions, such as hypertension [123] and heroin dependence [124]. Furthermore, the age at onset of PDP seems to play a significant role in the effect of *DRD* polymorphisms in PDP, but not all of the relative studies took this factor into consideration.

### 4.4. Genetic Polymorphisms of the Cholecystokinin System and PDP

Cholecystokinin (CCK) is a neuropeptide with a critical role in dopaminergic modulation, which is found in both the gastrointestinal and central nervous system (CNS). There are two types of CCK receptors (CCKRs), A and B (CCKAR and CCKBR, respectively); CCKRs are G protein-coupled receptors with varying affinity for CCK forms, including CCK4 and sulfated and unsulfated CCK8. CCK regulates dopamine release in the mesolimbic pathway, thereby modulating behavior. Dopamine and CCK also coexist in dopaminergic neurons [125], and dopamine can stimulate *CCK* gene expression [126]. In addition, decreased CCK8 immunoreactivity has been demonstrated in the SN of PD patients [127]. CCKAR is mainly implicated in the CCK-mediated dopamine release in the posterior nucleus accumbens, whereas CCKBR is responsible for the CCK-mediated inhibition of dopamine release in the anterior nucleus accumbens [128]. The *CCK* –45 locus C/T genotype has been more frequently found in PD patients compared to age-matched controls [129], although this finding has not been confirmed in other studies [130].

Given the fact that CCK is majorly implicated in dopamine-related behavior, it has been hypothesized that *CCK* gene polymorphisms could affect the risk for several psychiatric conditions. In this regard, it has been shown that *CCK* and *CCKR* polymorphism may be associated with panic disorder, alcoholism and delirium tremens, bipolar disorder and schizophrenia [131]. Given the molecular differences in PDP and schizophrenia pathogenesis, and the mainly auditory hallucinations in schizophrenia, these findings are not directly comparable with PDP. Therefore, it has been suggested that *CCK* and *CCKR* gene polymorphisms may be also related to PDP development, and several studies have investigated this relationship.

In this context, a study among Japanese subjects showed that TT and CT genotypes at −45 locus in the promoter region of the *CCK* gene have been associated with higher frequency of hallucinations in levodopa-treated PD patients [129]. Another study in the Chinese population indicated that the T allele in *CCK* −45 locus of the promoter region was associated with the presence of visual hallucinations in levodopa-treated PD patients [130]. In vivo evidence has demonstrated that the *CCK* −45C > T polymorphism does not affect gene transcription [132], suggesting that this gene polymorphism may not at least directly alter CCK levels [17]. It has been proposed that functional mutations in the *CCK* gene possibly being in a linkage disequilibrium with this polymorphism [130]. Moreover, although *CCKAR* (779 T > C) or *CCKBR* (1550 G > A) gene polymorphisms alone were not related with visual hallucinations in PD patients in this study, the co-presence of a *CCK* −45 locus T allele and a *CCKAR* C allele was significantly associated with a greater risk of visual hallucinations [130]. Based on this evidence, a synergistic effect of *CCK* and *CCKAR* gene polymorphisms has been proposed, suggesting that a potential interaction between these genetic factors could play a major role in the pathophysiology of PDP, at least in the Asian population.

Another study aiming to confirm these findings in a population of white ethnicity illustrated a similar pattern of association between the *CCK* T allele as well as the combination of *CCK* T and *CCKAR C* alleles and PDP occurrence, but the frequency of the *CCK* T allele was particularly low, not allowing for adequate power for statistically significant results [133]. A larger study also failed to find any significant associations between *CCK*, *CCKAR* and *CCKBR* gene polymorphisms and PDP [134]. Therefore, it has been suggested that the effect of the *CCK*-related gene polymorphisms on PDP risk largely depends on race and ethnicity [134].

### 4.5. Relationship between HOMER1 Gene and PDP

Apart from impaired dopaminergic neurotransmission, glutamatergic imbalance has been proposed to play a major role in PDP pathophysiology. In this context, metabotropic glutamate receptors (mGluRs) critically regulate synaptic activity and neuronal function, and they have also been related to drug-induced neuroplasticity in the nucleus accumbens [135]. The Homer family, encoded by *HOMER1*, *-2* and *-3* genes, is a group of postsynaptic density proteins interacting with the intracellular domain of mGluR1, thereby regulating several signaling pathways [136]. HOMER1 is highly expressed in the brain, and they are implicated in glutamatergic neurotransmission and synaptic plasticity [137]. Homer1a isoform is implicated in several neurological and psychiatric disorders, such as epilepsy, drug abuse and schizophrenia [138]. Homer1 knockdown can also protect dopaminergic neurons by modulating calcium homeostasis in in vitro models of PD [139]. In 6-hydroxydopamine (6-OHDA)-treated rat models of PD, deep brain stimulation of the subthalamic nucleus was associated with downregulation of *HOMER1*, potentially leading to decreased sensitivity to glutamate in basal ganglia [140]. Further in vivo evidence has revealed that Homer1a is overexpressed after the use of dopaminergic agonists [141], suggesting its possible role in treatment-related symptoms in PD.

The A variant in rs4704559 allele and the C variant in rs4704560 allele of the *HOMER1* promoter have been associated with higher risk of hallucinations in PD [142]. More specifically, carrying the A variant in rs4704559 allele of the promoter of the *HOMER1* gene was related with five times higher risk of developing hallucinations in PD in this study. Moreover, another study revealed a protective role of the rs4704559 G allele of the *HOMER1* promoter against the development of visual hallucinations in levodopa-treated Brazilian PD patients [137]. However, this study did not confirm the association between PDP and rs4704559 and rs4704560 of *HOMER1* as the previous study did. It has been hypothesized that these genetic polymorphisms may affect gene expression, although this assumption has not been tested [137,142].

### 4.6. Catechol-O-Methyltransferase (COMT) Gene Polymorphisms and PDP

Catechol-O-methyltransferase (COMT) plays a critical role in the metabolism of dopamine. It has been indicated that the *COMT* rs4680 genotype is associated with depressive symptoms [143] and the *COMT* Val158Met polymorphism is related to MDMA-induced psychotic symptoms [144].

No associations between *COMT* gene polymorphisms and PDP have been revealed in three studies [100,111,114]. In accordance, *COMT* Val158Met polymorphism was not related to psychotic manifestations in Lewy body dementias in another study, suggesting that *COMT* gene polymorphisms might not significantly affect PDP risk [145]. However, a recent study has shown that PD patients carrying at least one *COMT* rs165815 C allele displayed significantly less frequent levodopa-induced visual hallucinations compared to those not carrying this specific allele [115]. In addition, *COMT* haplotypes have been associated with the development of visual hallucinations in PD patients in this study.

Dopamine is produced in the dopaminergic neurons from levodopa by the enzyme dopa decarboxylase (DDC). Interestingly, *COMT-DDC* gene–gene interaction has been shown to affect the risk of levodopa-induced visual hallucinations in PD patients [115]. *DDC* rs921451 has been shown to reduce the enzyme activity, possibly resulting in lower dopamine levels [146]. Therefore, the effects of *COMT-DDC* interaction with PDP risk might explained by the alterations in dopamine concentrations [115].

### 4.7. Angiotensin I-Converting Enzyme and PDP

Emerging evidence highlights the role of angiotensin I-converting enzyme (ACE), which is the main enzyme of the renin-angiotensin system (RAS), in the pathogenesis of neurological and psychiatric disorders. The D allele of *ACE* has been associated with psychotic symptoms in bipolar disorder, whereas the I allele may protect against the development of schizophrenia and bipolar disorder [147]. In PD, dopaminergic treatment has been shown to affect ACE levels in the cerebrospinal fluid (CSF) [148], and perindopril,-an ACE inhibitor- treatment, may improve motor symptoms in levodopa-treated PD patients [149]. A very recent study has also shown that angiotensin II receptor blockers with blood—brain barrier penetrating capacity reduce the risk for PD among patients with ischemic heart disease [150]. *ACE* gene polymorphisms have been demonstrated to increase the risk for PD [151], and the rs4646994 polymorphism of *ACE* has been recently associated with impulsive control disorder in PD patients [152].

*ACE-II* genotype has been associated with higher risk for levodopa-induced psychotic manifestations in Chinese PD patients [153]. On the contrary, no relationship between PDP and *ACE-II* gene polymorphisms was replicated in a study of Italian PD patients [154], suggesting that racial or ethnic differences may account for these conflicting results.

### 4.8. ANKK1 Gene Polymorphisms and PDP

Ankyrin repeat and kinase domain containing I *(ANKK1)* gene polymorphisms have been associated with alcoholism and other neuropsychiatric disorders, including PD [155]. The GG rs2734849 *ANKK1* gene polymorphism has been associated with the development of PDP in levodopa-treated PD patients in another recent study [111]. rs2734849 polymorphism is in the coding region of *ANKK1* gene and it is able to regulate DRD2 density by changing NF-κB expression levels [156].

### 4.9. Serotoninergic System Genes and PDP

Serotoninergic dysfunction plays a critical role in PD pathophysiology. A significant serotoninergic neuronal loss is observed in the raphe nucleus of the brainstem, accompanied by reduced serotoninergic terminals in the putamen, frontal and temporal cortex [157]. A repeat polymorphism of the promoter region of the serotonin transporter (*5-HTT*) gene, 5-HTTLPR, has been associated with depression and anxiety in PD patients in some —but not all—studies [158,159,160]. In addition, 5-HT2A receptor gene polymorphisms have been associated with negative symptoms in schizophrenia [161] and psychotic manifestations in AD [162]. Pimavanserin, a 5-HT2AR inverse agonist and antagonist, has been approved by the FDA for PDP, further strengthening the hypothesis that serotoninergic system is critically implicated in PDP [163].

In this context, no associations were observed between the 5-HT2A receptor T102C polymorphism or the 5-HTTLPR and the presence of visual hallucinations in non-demented PD patients [164]. Another study has demonstrated that the L/L genotype of the *5-HTTLPR* gene was associated with delusions in PD patients with dementia as well as patients with dementia with LBs [165]. The diverse characteristics of the included patients in these studies may explain their different results, since the second study included patients with synucleinopathy with dementia (either PD dementia or dementia with LBs) [17].

### 4.10. Microtubule Associated Protein Tau (MAPT) Gene Polymorphisms and PDP

As mentioned above, the *MAPT* gene exists in three genotypes (H1/H1, H1/H2 and H2/H2). A postmortem study showed that PD cases with the H1/H1 genotype had significantly more frequently hallucinations compared to non-carriers, independent of the disease stage [101]. However, no link was revealed between *MAPT* gene polymorphisms and psychotic manifestations in PD patients in another study [16].

### 4.11. SNCA Gene Polymorphisms and PDP

No association has been found between psychotic symptoms and genetic polymorphisms in the *SNCA* gene [16]. In particular, the *SNCA*-REP1 261 allele, which has been shown to increase the risk for PD [166], was not associated with psychotic manifestations in PD patients [16].

### 4.12. IL-6 Gene Polymorphisms and PDP

Neuroinflammation is one of the core pathophysiological mechanisms underlying PD pathogenesis. It has been proposed that it may contribute to the development of non-motor symptoms of PD, although its specific role in PDP still remains elusive [167]. Increased plasma interleukin-6 (IL-6) levels were identified as a major contributor to mortality in PD patients [168]. In addition, elevated plasma CRP levels were associated with the occurrence of hallucinations or illusions in PD patients, suggesting that excessive systemic inflammation might increase the risk for PDP [169].

A recent study aimed to simultaneously assess the effect of 34 genetic and 16 clinical variables on the development of visual hallucinations in PD patients, via the estimation of a clinical-pharmacogenetic index [170]. The genetic factors were selected based on the existing literature and the major cellular pathways implicated in PD and PDP, including dopamine neurotransmission and metabolism, inflammation, oxidative stress and neurodevelopment [170]. This study demonstrated that *IL-6* rs1800795 gene polymorphism was associated with lower risk of visual hallucinations in PD patients [170]. Given the fact that this polymorphism has been shown to reduce IL-6 expression levels [171], it can be speculated that neuroinflammation may play a critical role in PDP.

### 4.13. GPX1 Gene Polymorphisms and PDP

Oxidative stress plays a critical role in PD pathophysiology, and lower levels of glutathione peroxidase-1 (GPX1), an enzyme with anti-oxidant activity, have been shown in the SNpc of PD patients [172]. *GPX-1* polymorphisms rs1050450 and rs1800668 have been associated with schizophrenia in the Chinese population [173], suggesting a potential role of *GPX-1* gene in psychosis.

In this context, *GPX1* rs1050450 gene polymorphism has been recently associated with higher frequency visual hallucinations in patients with PD [170]. This polymorphism may reduce the activity of this enzyme, leading to a more weakened defensive mechanism against oxidative stress [171], which provides a possible underlying molecular mechanism.

### 4.14. MAOB Gene Polymorphisms and PDP

*MAOB* gene polymorphisms have been related to the development of schizophrenia [174,175]. *MAOB* rs1799836 polymorphism has been shown to protect against PDP development in a recent study [170]. It has been proposed that this effect may be due to the lower turnover of dopamine in carriers of the G allele [176].

### 4.15. BIRC5 Gene Polymorphisms and PDP

It has been shown that *BIRC5* rs8073069 polymorphism may lower the risk of the development of visual hallucinations in PD patients [170]. One potential explanation might be the subsequent higher expression of survinin, which inhibits apoptosis, neuroinflammation and oxidative stress [177].

### 4.16. BDNF Gene Polymorphisms and PDP

A recent preclinical study demonstrated that chronic methamphetamine use may interact with brain-derived neurotrophic factor (*BDNF*) Val66Met to remodel psychosis-related pathways in the mesocorticolimbic circuitry [178]. The same polymorphism has been associated with the onset age of schizophrenia [179]. The Met allele has been associated with cognitive impairment in patients with PD [180]. This substitution can reduce the extracellular release of BDNF [181], resulting in its decreased availability for the neuronal cells.

In this context, it has been shown that *BDNF* G196A polymorphism was not associated with any non-motor symptoms in PD, including psychiatric manifestations [182]. However, a recent study has shown that *BDNF* Val66Met polymorphism was associated with mild behavioral impairment (MBI) in PD patients. In particular, compared to Val carriers, Met carriers displayed higher impairment in the mood/anxiety and psychotic subdomains [183], suggesting that Met polymorphism may increase the risk for PDP. Further studies are needed in order to elucidate the role of *BDNF* polymorphisms in PDP.

## 5. Potential Gene-Related PDP Therapeutic Implications

The response to a pharmaceutical agent depends on several factors related to pharmacokinetics and pharmacodynamics, including absorption, metabolism, distribution, elimination, blood–brain barrier permeability, etc. These factors are affected by both genetic polymorphisms and environmental exposures. The main goal of pharmacogenetics is to develop personalized treatment for each patient, according to genetic factors that may influence drug response, regarding efficacy and safety [184]. For instance, especially high doses of dopaminergic agonists could be avoided in PD patients carrying gene alleles strongly associated with higher risk of PDP development [122]. Interestingly, a randomized controlled trial demonstrated that donepezil treatment was associated with lower occurrence of psychosis only in PD patients not carrying the *APOE* ε4 allele [185], suggesting that *APOE* genotype may at least partially account for differences in the clinical response to acetylcholinesterase inhibitors among PD patients, in regard to psychotic symptoms. Furthermore, *DRD3* polymorphisms have been related to diverse clinical responses to olanzapine treatment in patients with schizophrenia [186], and this relationship might be of relevance to PD patients too.

A very recent study also demonstrated that the administration of a cholecystokinin analogue in 1-Methyl-4-Phenyl-1,2,3,6-Tetrahydropyridine (MPTP)-induced mouse models of PD led to increased motor behavior, dopaminergic neuronal survival and reduced neuroinflammation [187]. Given the important role of *CCK* polymorphisms in PD, this agent could be also explored against PDP. Additionally, an in vitro study has shown that SKF-96365, a non-specific store-operated calcium entry (SOCE) inhibitor, may protect PC12 cells against MPP+-induced toxicity, by altering Homer1-mediated calcium release [188]. Since *HOMER1* gene polymorphisms may affect PDP risk, this agent could be investigated especially in *HOMER1*-related PDP cases.

As abovementioned, pimavanserin, a highly selective and well-tolerated 5-HT2A receptor inverse agonist, is the first FDA-approved pharmaceutical agent for the treatment of PDP. Pimavanserin has been shown to ameliorate psychotic manifestations in PD without worsening motor symptoms [189]. Although no associations have been identified between the *5-HT2A* receptor gene polymorphism and visual hallucinations in PD patients [164], larger studies investigating the potential role of this gene in the clinical response to pimavanserin might be of value.

## 6. Conclusions and Future Perspectives

Although the association between several genetic factors and PDP has been investigated in several studies (summarized in Table 2), apart from cholecystokinin system and possibly *APOE* gene polymorphisms, most of them have shown conflicting results. The −45 C > T *CCK* gene polymorphism has been associated with hallucinations in PD in most related studies, and its pathophysiological role in PDP should be further explored for the development of targeted therapeutic approaches.

The partially conflicting results of the abovementioned studies could be explained by different methodological designs. The type of study design (retrospective or prospective) is of paramount importance, as well as the consideration of disease duration and age at onset of the disease. Given the higher risk of PDP as the disease progresses, not taking into account age and disease duration, many cases of PDP could have been missed. For instance, *APOE4* seems to be associated with earlier onset of PDP. Longitudinal studies during the whole disease course would also be necessary for the elucidation of these contradictory results. In addition, given the well-known association between dopaminergic therapy and dementia and PDP, it would be very important in future studies to analyze gene–PDP relationships after adjustment for the presence of these two factors. This is particularly important for genetic factors already known to possibly affect PD-related dementia, including *APOE4* allele or *DRD* gene polymorphisms [190]. As abovementioned, another important consideration is the definition of PDP among studies. Due to relatively small sample sizes and lack of relevant data in the relevant studies, psychotic symptoms have been considered as dichotomous variables (yes/no), not taking into account the severity of PDP via quantitative measures. Several studies have included severe PDP cases which required pharmacological treatment, so PD cases with minor psychotic symptoms might have been missed. However, other studies have included patients with PDP regardless of severity. Given the fact that PDP is often considered a continuum, with minor symptoms progressing to hallucinations with insight and then to hallucinations without insight and delusions [191], it is important to include PD patients with minor phenomena too in future studies. In addition, the type of medical center from which data were collected is also important for potential selection bias which may significantly affect the results; in academic centers or movement disorder units, PD patients with atypical symptoms, earlier age at onset or difficult management are more likely to be examined. On the other hand, PD patients with more severe cognitive impairment, psychiatric symptoms or functional disability would be more likely to visit these centers. Therefore, these samples may not be representative of the general PD population. Ethnic and racial factors may also contribute to the discrepancies of the abovementioned studies, as already discussed above. Since most relative studies were based on clinical diagnosis of PD, autopsy confirmation would be also very helpful in future studies.

Most studies have investigated the relationship between genetic factors and visual hallucinations in PD. Since the inclusion of PD patients with delusions would result in phenotypic heterogeneity of the participants in these studies, it would be helpful to separately explore associations between delusions and genetic polymorphisms in PDP. Although non-visual hallucinations and delusions are much more uncommon and often co-exist with visual hallucinations [17], the clinical heterogeneity of PDP might be due to a distinct underlying genetic landscape [153].

Importantly, most studies discussed herein have examined the potential relationship between the most common and well-studied gene polymorphisms with PDP. Hence, the association between other polymorphisms in the same genes and PDP development might have been missed. In this regard, the examination of other genetic predictors in GWAS would be very helpful.

Interestingly, impaired network connectivity has been demonstrated in PD patients with hallucinations; a recent study indicated that regional gene expression in regions of the disturbed subnetwork in PD patients with visual hallucinations was characterized by downregulation of genes related to chromosome organization, mRNA metabolism, histone lysine methylation and upregulation of genes related to protein targeting and intracellular and membrane localization [192]. In this context, these genes could represent potential candidates for further research regarding PDP susceptibility.

Another important note is the fact that the abovementioned studies have investigated the relationships between genetic factors and psychotic symptoms in idiopathic PD. However, it would be of great interest to explore potential links between genetic factors predisposing one to PDP in genetic forms of PD. In this context, Parkin has been shown to regulate glutamatergic AMPA receptor trafficking by binding to Homer1, and mutant forms of Parkin display disrupted binding capacity [193]. Although Parkin-related PD is not associated with specific psychiatric manifestations [194], Homer1-Parkin interactions may possibly alter the risk of psychotic symptoms specifically in these patients. Furthermore, *DJ-1* could transcriptionally activate *CCK* by binding to the RRE-binding protein 1 (RREBP1) [195]. PD patients carrying *DJ-1* mutations present with early-onset motor impairment followed by psychotic manifestations among other non-motor symptoms [196]. Hence, CCK-related pathways might be implicated in PD cases caused by *DJ-1* mutations and could be further explored.

Other potential genetic candidates for PDP could be those related to both PD and schizophrenia. A good example is *VMAT2*; two polymorphisms of this gene (rs363371, rs363324) have been associated with PD risk in the Italian population [197], and this gene has been also show to affect the risk of schizophrenia in another study [198]. Therefore, the role of *VMAT2* polymorphisms in PDP should be further examined.

As abovementioned, the clinical characteristics of PDP have a different pattern compared to other diseases with psychotic symptoms such as schizophrenia or bipolar disorder. For example, PD patients mainly experience visual and not auditory hallucinations—which predominate in schizophrenia. Some of the most well-studied genetic factors in schizophrenia are *ABT1*, *AKT1*, *ARVCF*, *BTN3A1*, *BTN3A2*, *CACNA1C*, *CENTG2*, *CHRNA7*, *COMT*, *CNTNAP2*, *DISC1*, *DRD2*, *DRD4*, *DTNBP1*, *EVI*, *FAM69A*, *FMR1*, *FXR1*, *HMGN4*, *NOTCH4*, *NRG1*, *RPL5* and *TCF4* [199]. Furthermore, *ODZ4*, *ANK3*, *SYNE1*, *TRANK1*, *LMAN2L* and *PTGFR* genes have been related with bipolar disorder [200]. In addition, higher genetic risk of schizophrenia has been associated with psychotic symptoms in bipolar disorder [201]. However, most of the genes that have been related to schizophrenia are different from those related to PDP, possibly reflecting the diversity in the clinical phenotype of these conditions.

It is well-established that smoking protects against PD development [202,203]. The *DRD2* gene has been associated with an increased risk for smoking [204], and smoking has been recently shown to be related with PDP [204]. Therefore, a potential interaction between smoking, *DRD2* polymorphisms and PDP should be further explored. *CCK* and *MAO-B* genes have been associated with smoking behavior as well [205,206]. In addition, coffee consumption has been associated with visual hallucinations in levodopa-treated PD patients [170]. Given the fact that *COMT* gene polymorphisms may influence the effects of coffee on other diseases [207], the role of COMT-coffee interaction in PDP development should be also examined.

Regarding prognosis, given the fact that PDP leads to higher morbidity and mortality, a personalized plan for nursing and medical needs would be beneficial for PD patients at increased risk for PDP [122].

**Table 2:** Summary of main genes related to PDP development.

Gene	Protein Function	Main Findings	Number	Age at Onset, Years	Sex (m/f)	Reference
*APOE*	Transport of lipids in the brain	- The ε4 allele associated with earlier onset of hallucinations in PD, mainly at the early stages of the disease	105	63.1 ± 10.4	56/49	[98]
			87	60.1 ± 12.7	53/34	[102]
			424	50–80	317/107	[103]
*DAT*	Regulation of dopamine levels through its reuptake in neurons	- The 40-bp VNTR associated with psychosis in levodopa-treated PD patients only in Caucasians	183	67.0 ± 10.6	107/76	[109]
		- The −839 C > T allele in the 5′ UTR associated with visual hallucinations in levodopa-treated PD patients	196	59.2 ± 11.0	100/96	[112]
		- The 10/11, 10/8 and 10/9 genotypes of rs28363170 associated with visual hallucinations in Brazilian PD patients	224	56.2 ± 10.2	129/95	[114]
*DRD*	Receptor of dopamine	- *DRD1*: −48GG and 62TT alleles in PD patients associated with higher risk for visual hallucinations with an earlier onset	84	64.0 ± 8.7	44/40	[122]
		- *DRD2*: C allele of the TaqIA polymorphism more frequently present in PD patients with late-onset hallucinations	84	52.88 ± 12.52	50/34	[117]
		- *DRD2*: the TT rs6277 variant associated with PDP in levodopa-treated PD patients	234	58.3 ± 8.1	133/101	[111]
		- *DRD3*: Ser9Gly polymorphism associated with the severity of depressive symptoms in PD patients	61	49–88	41/20	[121]
		- *DRD3*: Ser/Ser and Ser/Gly genotypes of rs6280 associated with higher frequency of visual hallucinations in Brazilian population	224	56.2 ± 10.2	129/95	[114]
		- *DRD3*: rs6280 C allele associated with levodopa-induced visual hallucinations	231	54.8–71.7	132/99	[115]
		- *DRD4*: CG allele of rs747302 associated with later onset of hallucinations as compared to *DRD4* CC alleles	84	64.0 ± 8.7	44/40	[122]
*CCK*	Regulation of dopamine in the CNS	- TT and CT genotypes associated with higher frequency of hallucinations in levodopa-treated PD patients	116	68.2 ± 9.2	48/68	[129]
		- T allele at −45 locus in combination with C allele of 779T > C variant in the receptor encoding gene (*CCKAR*) associated with a greater risk of visual hallucinations	160	58.8 ± 12.6	89/71	[130]
*HOMER1*	Regulation of glutamatergic neurotransmission and synaptic plasticity	- Protective role of rs4704559 G allele in the promoter against the development of visual hallucinations in levodopa-treated Brazilian PD patients	205	58.77 ± 11.07	105/100	[137]
		- Rs4704559 A allele and rs4704560 C allele in the promoter associated with higher risk of hallucinations in PD	131	56.4 ± 8 9.6	65/66	[142]
*COMT*	Metabolism of dopamine	- Rs165815 C allele associated with low frequency of levodopa-induced visual hallucinations	231	54.8–71.7	132/99	[115]
*ACE*	Regulation of blood pressure and electrolyte balance	- Rs4646994 polymorphism associated with impulsive control disorder in PD patients	49	65.8 ± 8	23/26	[152]
		- *ACE-II* genotype associated with higher risk for levodopa-induced psychotic manifestations in Chinese PD patients	251	63.3 ± 11.4	132/119	[153]
*ANKK1*	Maintenance and/or functioning of dopaminergic pathways	- Rs2734849 GG genotype associated with the development of PDP in levodopa-treated PD patients	234	58.3 ± 8.1	133/101	[111]
*5-HTT*	Transporter of serotonin	- S allele of the *5-HTTLPR* polymorphism associated with anxiety and depression in PD patients	32	67.0	20/12	[159]
		- S allele of the *5-HTTLPR* polymorphism associated with depression in PD patients	72	n/a	39/33	[160]
		- The L/L genotype of the *5-HTTLPR* polymorphism associated with delusions in demented PD patients	87	75.9 ± 6.4	n/a	[165]
*MAPT*	Promotion of microtubule assembly and stability	- H1/H1 genotype frequently associated with hallucinations as compared to non-carriers, independently of disease stage	72	65.9	53/19	[101]
*SNCA*	Regulation of synaptic vesicle trafficking and neurotransmitter release	- No variants associated with psychotic manifestations in PD patients	63	63.7–72.9	36/27	[16]
*IL-6*	Pro-inflammatory cytokine	- Rs1800795 polymorphism associated with lower risk of visual hallucinations in PD patients	214	61.7	124/90	[170]
*GPX1*	Anti-oxidant enzyme	- Rs1050450 polymorphism associated with higher frequency visual hallucinations in PD patients	214	61.7	124/90	[170]
*MAOB*	Metabolism of biogenic amines	- Protective role of rs1799836 polymorphism against PDP development	214	61.7	124/90	[170]
*BIRC5*	Inhibition of apoptosis	- Rs8073069 polymorphism associated with lower risk of the development of visual hallucinations in PD patients	214	61.7	124/90	[170]
*BDNF*	Regulation of the survival and growth of neurons	- Met variant of Val66Met polymorphism associated with higher impairment in the mood/anxiety and psychotic subdomains in PD patients	136	69.2 ± 8.1	82/54	[183]

ACE, angiotensin I-converting enzyme; ANKK1, ankyrin repeat and kinase domain containing I; APOE, apolipoprotein E; BDNF, brain-derived neurotrophic factor; BIRC5, baculoviral IAP repeat containing 5; CCK, cholecystokinin; CCKAR, cholecystokinin A receptor; CNS, central nervous system; COMT, Catechol-O-methyltransferase; DAT, dopamine transporter; DRD, dopamine receptor; GPX1, glutathione peroxidase-1; IL-6, interleukin-6; MAOB, monoamine oxidase B; MAPT, microtubule-associated protein tau; n/a, not available; PDP, psychosis in PD; SNCA, α-synuclein.

## Figures and Tables

**Table 1:** Established Parkinson’s disease-causing genes and risk factors.

Gene	Function	Main Types of Mutations/Variants
**Autosomal dominant**		
*SNCA*	Synaptic vesicle trafficking and neurotransmitter release	Genomic multiplications (duplications, triplications) and missense mutations
*LRRK2*	Neuronal vesicular trafficking and autophagic protein degradation	Missense mutations
*VPS35*	Retromer and endosomal trafficking	Missense mutations
**Autosomal recessive**		
*PRKN*	Mitochondrial homeostasis	Structural variants (genomic multiplications and deletions in exons or gene promoter), missense, nonsense, splice-site and frameshift mutations
*PINK1*	Mitochondrial homeostasis	Structural variants, missense, nonsense and frameshift mutations
*DJ-1*	Mitochondrial homeostasis	Deletions, missense and frameshift mutations
**Risk factors**		
*SNCA*	Synaptic vesicle trafficking and neurotransmitter release	Polymorphic variants, often in non-coding regions
*LRRK2*	Neuronal vesicular trafficking and autophagic protein degradation	Polymorphic variants
*MAPT*	Microtubules assembly and stabilization	Polymorphic variants
*GBA*	Lysosomal	Biallelic (homozygous or compound heterozygous) mutations
**X-linked**		
*RAB39B*	Vesicular trafficking	Whole gene deletion, missense, splicing and frameshift variants

DJ-1, protein deglycase; GBA, glucosylceramidase beta; LRRK2, leucine-rich repeat kinase 2; MAPT, microtubule-associated protein tau; PINK1, PTEN-induced putative kinase 1; PRKN, parkin; RAB39B, Ras Analogue in Brain 39b; SNCA, α-synuclein; VPS35, vacuolar sorting protein 35.

## Data Availability

Not applicable.
